# Unexplained Intraoperative Movement During a Complex Craniotomy for Recurrent Petrous Apex Cholesterol Granuloma: A Case Report

**DOI:** 10.7759/cureus.70660

**Published:** 2024-10-01

**Authors:** Lamis Kattan

**Affiliations:** 1 Anesthesia and Critical Care, King Abdulaziz University Faculty of Medicine, Jeddah, SAU; 2 Anesthesia and Critical Care, McMaster University, Hamilton, CAN

**Keywords:** cholesterol granulomas, cranial nerve dysfunction, depth of anesthesia, neuromonitoring, patient safety, petrous apex, seizures, total intravenous anesthesia (tiva)

## Abstract

Intraoperative patient movement under general anesthesia, even with multiple monitoring modalities and adequate anesthetic depth, is rare but can lead to serious complications. Such movements are particularly dangerous in neurosurgical procedures, where precision is crucial. Similar risks exist in ophthalmic, spinal, and cardiac surgeries, where patient immobilization is vital to prevent adverse outcomes.

This report examines the case of a 37-year-old male diagnosed with recurrent cholesterol granuloma located at the petrous apex, which necessitated neurosurgical intervention. During the procedure, the patient was placed under deep general anesthesia, and multiple neuromonitoring techniques were used to track neural and motor activity. Despite maintaining stable hemodynamic parameters and unremarkable neuromonitoring results, the patient suddenly exhibited abrupt, forceful movements involving his head and upper arms. This unexpected event during a delicate neurosurgical procedure posed a significant challenge, prompting a deeper investigation into the possible underlying causes of the patient's sudden movements, which could include factors such as insufficient anesthetic depth, muscular or neural irritation, seizure activity, or mechanical factors related to surgical equipment or technique.

This case highlights the critical role of comprehensive intraoperative monitoring in ensuring patient safety, particularly during complex neurosurgical procedures where precision is essential. The use of total intravenous anesthesia (TIVA), as was used in this case, presents unique challenges, as it requires a careful balance of maintaining adequate anesthetic depth without interfering with the neuromonitoring signals used during the procedure to ensure neural integrity.

## Introduction

Although rare, intraoperative patient movement can compromise the safety and success of delicate and complex surgeries such as neurosurgery, where even small disruptions can result in significant complications. This case report explores possible explanations for such a rare occurrence in a patient undergoing complex neurosurgery, where sudden movement occurred despite stable anesthetic parameters. This case raises critical questions about the causes and management of such dangerous events. Intraoperative patient movement during surgery, with the use of rigid head immobilization devices such as Mayfield skull clamps, presents critically significant clinical risks. Mayfield pins are used to stabilize the skull during neurosurgical procedures; however, complications may arise from poor fixation or intraoperative movement. Among these complications are depressed skull fractures, which could arise if the pins were placed in sites where the cranial bone is thinner or if too much force was used. Other possible complications include slippage of the clamp, which may cause the loss of head stability and, consequently, trauma, and vascular insults, which include epidural hematoma and venous air embolism, in cases of improper or prolonged application of pins. However, the risk of cervical spine injury or strain due to abrupt patient movement is the complication most relevant to this case report [1,2].

Cholesterol granulomas are benign granulomatous lesions of the middle ear that can also affect the petrous apex or the mastoid [3]. They usually occur in young adults with a history of middle ear trauma or chronic infections. Despite their benign nature, these rapidly growing lesions can lead to significant clinical symptoms due to their location and potential to compress adjacent neural structures. The anesthetic management of these cases presents an extra layer of complexity, particularly when precise neuromonitoring modalities are employed to assess neural integrity, which precludes the use of muscle relaxants during the procedure beyond the induction dose [4].

## Case presentation

A 37-year-old male who was otherwise fit and healthy presented with a recurrence of his right-sided petrous apex cholesterol granuloma, which was previously successfully resected. His current presentation caused complete right-sided hearing loss, diplopia, tinnitus, dizziness, and nocturnal seizures. The patient had no other medical conditions and had not experienced any other significant health changes since his initial surgery.

Preoperatively, while on the standard Canadian Anesthesia Society (CAS) monitors, we administered 2 mg of midazolam intravenous (IV) for anxiolysis [5]. The patient was then transported to the operating room with standard monitors. General anesthesia was induced using 150 mcg of fentanyl IV, a total of 300 mg propofol IV, remifentanil 40 mcg, and rocuronium 50 mg IV, followed by an uneventful and successful endotracheal intubation and arterial line placement. The patient was then maintained on TIVA using propofol 100-120 mcg/kg/minute and remifentanil 0.05-0.2 mcg/kg/minute and following the "Eleveld 2.0" target-controlled infusion (TCI) model [6]. A bilateral scalp block was done using sterile techniques for postoperative pain management. Dexamethasone 8 mg and cefazolin 2 g were administered before the surgical incision. Intraoperatively, the blood pressure goal consisted of a mean arterial pressure range of 70-90 mmHg.

Prior to a multidisciplinary surgical team, consisting mainly of neurosurgery and otolaryngology surgeons, proceeding with the surgery, padded bolsters were used to position the patient supine with a slight leftward tilt carefully. His head was secured using the three-point Mayfield fixation frame, maintaining a neutral and slightly flexed neck following the middle cranial fossa approach [7]. Neurophysiologists placed pin electrodes to monitor electroencephalogram (EEG) waveforms, somatosensory evoked potentials (SSEP), and motor evoked potentials (MEP) of the right facial and trigeminal cranial nerves [8,9]. After the surgical team concluded their check of the neuro-navigation system, a processed electroencephalogram (EEG) device was designed to evaluate the patient's consciousness level while under anesthesia; entropy was applied by the anesthesia team [10].

The initial phases of the surgery were uneventful, as reflected by stable hemodynamic parameters, including a heart rate averaging 68 beats per minute and mean arterial pressure readings between 80 and 95 mmHg. EEG monitoring showed predominantly delta (0.5-4 Hz), alpha (8-12 Hz), and occasional theta (4-7 Hz) waveforms. However, as the surgical team approached the critical stage of resecting the part of the lesion that was adherent to the facial nerve, the patient unexpectedly exhibited strong head and upper limb movements, despite being under sufficiently deep general anesthesia and immobilized by Mayfield pins. Entropy values remained within the target range of 40-60 throughout much of the operation, with an average of 42 shortly before the movement, indicating an appropriate depth of anesthesia. These sudden movements presented significant risks to both the surgical procedure and the patient's safety.

**Figure 1:**
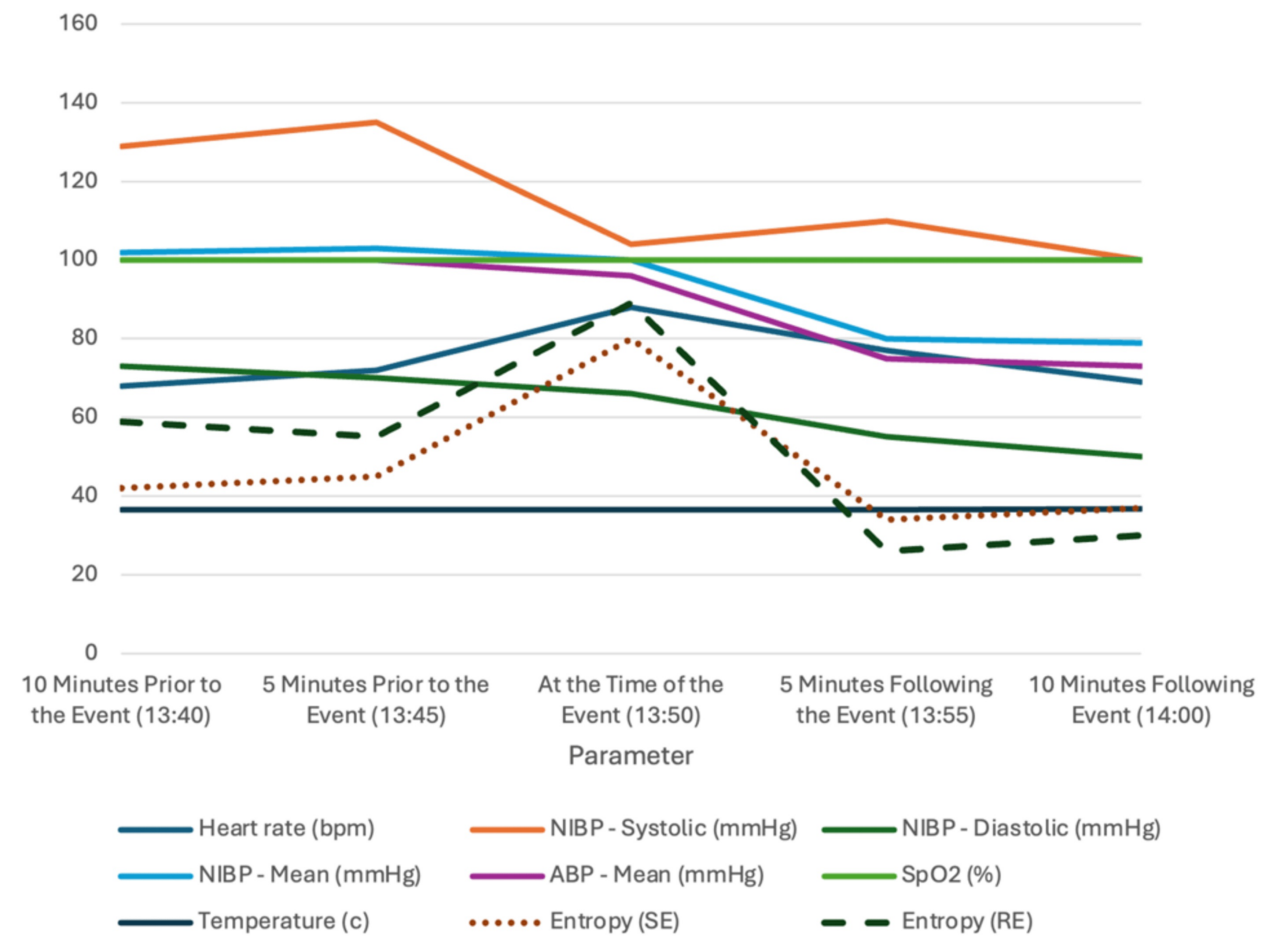
Graphical Representation of the Hemodynamic and Physiological Parameters Before, During, and After the Event bpm, beats per minute; NIBP, noninvasive blood pressure; ABP, intra-arterial blood pressure; SpO_2_, oxygen saturation; SE, state entropy; RE, response entropy

In response, the surgical team quickly restrained the patient to prevent further complications, while the anesthesia team swiftly maintained the patient's vital signs and appropriate depth of anesthesia. A bolus of 50 mg of propofol and 2 mg of midazolam was given initially, as the most likely diagnosis at the time was a seizure activity [11,12]. A rapid yet thorough equipment check for potential malfunction was done while ensuring the consistent delivery of total intravenous anesthesia (TIVA) through properly functioning vascular access to exclude any other possible causes of the event. A loading dose of levetiracetam 1 g was given, and the propofol and remifentanil infusions were adjusted as needed. The patient was confirmed stable, as evidenced by the post-intervention entropy values dropping to 30s. Following the multidisciplinary team confirming the patient's stability, with a heart rate of 77 beats per minute and blood pressure at 110/55 mmHg, the operation resumed smoothly, demonstrating effective teamwork and communication in handling the intraoperative event (Table 1 and Figure 1).

**Table 1:** Hemodynamic and Physiological Parameters Before, During, and After the Event bpm, beats per minute; NIBP, noninvasive blood pressure; ABP, intra-arterial blood pressure; SpO_2_, oxygen saturation; SE, state entropy; RE, response entropy

Parameter	10 Minutes Prior to the Event (13:40)	5 Minutes Prior to the Event (13:45)	At the Time of the Event (13:50)	5 Minutes Following the Event (13:55)	10 Minutes Following the Event (14:00)
Heart Rate (bpm)	68	72	88	77	69
NIBP, Systolic (mmHg)	129	135	104	110	100
NIBP, Diastolic (mmHg)	73	70	66	55	50
NIBP, Mean (mmHg)	102	103	100	80	79
ABP, Mean (mmHg)	100	100	96	75	73
SpO_2_ (%)	100	100	100	100	100
Temperature (°C)	36.6	36.6	36.6	36.6	36.7
Entropy (SE)	42	45	80	34	37
Entropy (RE)	59	55	89	26	30

A comprehensive evaluation was conducted during the postoperative period, thoroughly examining the intraoperative data and monitoring records. This detailed review revealed no indications of inadequate anesthesia depth or heightened brain activity to explain the patient's movements during the operation. The patient also underwent a thorough physical examination, which confirmed no memory of awareness under general anesthesia during the procedure. This revealed only a minor skin injury at one of the Mayfield pin sites, which was sutured and bandaged appropriately. He was otherwise awake, alert, oriented, and experiencing adequate pain control.

## Discussion

Intraoperative movement during neurosurgery, particularly under neuroanesthesia, can have serious consequences for surgical outcomes. Such movement may disrupt critical neuromonitoring techniques, such as motor evoked and sensory evoked potentials (MEPs and SSEPs), which are key for detecting early neurological issues. Inadequate anesthesia or patient movement can compromise neurophysiological readings, heightening the risk of injury or failed interventions. Thus, maintaining stable anesthesia and immobility is crucial for ensuring surgical precision and optimal patient safety [1,2].

This case illustrates the challenges of managing intraoperative instability in complex neurosurgery. Despite stable anesthetic parameters, the patient's unexpected movement highlights the limitations of current monitoring systems in detecting subtle neuromuscular or cerebral changes.

While intraoperative EEG and entropy readings showed appropriate anesthesia depth, transient lapses cannot be fully ruled out. Variations in anesthetic distribution or individual pharmacokinetics could lead to brief, undetected periods of inadequate anesthesia, which might not be immediately noticeable [13,14]. In this case, the patient's entropy dropped from 45 pre-event to 34 post-event, without any prior fluctuations, indicating that subtle inadequacies could have been missed by the monitoring equipment [11].

The movement may have been due to a neuromuscular reflex, influenced by positioning or environmental factors. Reflexive responses are rare under general anesthesia, but stimuli such as cranial nerve irritation can still elicit such movements. The patient had a history of nighttime seizures managed with levetiracetam. Though intraoperative seizures are uncommon when appropriate seizure-control measures are in place, surgical stress or neural irritation could trigger a seizure under anesthesia [8].

Intraoperative EEG showed no typical seizure signs before the movement. However, subclinical seizures or focal seizures not captured by standard EEG monitoring could explain the sudden movement. Similar cases of intraoperative seizures during craniotomy have been documented, underscoring the importance of vigilant monitoring [11]. The lack of EEG detection in these cases highlights the need for multi-modal monitoring strategies [13,14]. Studies have shown that elevated bispectral index values may indicate intraoperative seizures, emphasizing the limitations of single-modality monitoring and the importance of multi-modal approaches for complex neurosurgical procedures [12].

Comparable instances of intraoperative seizures during craniotomy under general anesthesia have been reported in the literature, reinforcing the need for careful monitoring [8]. Intraoperative refractory status epilepticus related to propofol use has also been reported, further highlighting the value of multi-modal monitoring in identifying and managing such events [13,14]. Studies have also detected intraoperative seizures through elevated bispectral index values during posterior fossa surgeries, supporting the need for multi-modal monitoring [12].

## Conclusions

Anesthetic management during complex neurosurgical procedures can be difficult due to multiple factors, namely, the need for a stable and steady surgical field. The potential for patient movement combined with the need to use rigid fixation devices such as the Mayfield pin system, while requiring little to no muscle relaxation for neuromonitoring purposes, can be severely hazardous. This case highlights the importance of careful monitoring and suggests that incorporating several different monitoring modalities could be necessary to guarantee patient safety. Moreover, enhancing available processed EEG devices and hemodynamic monitors may help avoid significant intraoperative complications. Future research should focus on identifying and validating these tools to improve patient safety and outcomes in neurosurgery.
